# Cosmopolitan inversions have a major impact on trait variation and the power of different GWAS approaches to identify associations

**DOI:** 10.1371/journal.pgen.1012012

**Published:** 2026-01-05

**Authors:** Benedict Adam Lenhart, Alan O. Bergland

**Affiliations:** Department of Biology, University of Virginia, Charlottesville, Virginia, United States of America; UC Santa Cruz, UNITED STATES OF AMERICA

## Abstract

The ability of genomic inversions to reduce recombination and generate linkage can have a major impact on genetically based phenotypic variation in populations. However, the increase in linkage associated with inversions can create hurdles for identifying associations between loci linked to inversions and the traits they impact. Therefore, the role of inversions in mediating genetic variation of complex traits remains to be fully understood. This study uses the fruit fly *Drosophila melanogaster* to investigate the impact of inversions on trait variation. We tested the effects of common inversions among a diverse assemblage of traits including aspects of behavior, morphology, and physiology, and identified that the cosmopolitan inversions In(2L)t and In(3R)Mo are associated with many traits. We compared the ability of different approaches of accounting for relatedness and inversion presence during genome-wide association to identify signals of association with SNPs. We report that commonly used association methods are underpowered within inverted regions, while alternative approaches such as leave-one-chromosome-out improve the ability to identify associations. In all, our research enhances our understanding of inversions as components of trait variation and provides insight into approaches for identifying genomic regions driving these associations.

## Author summary

Genomic inversions are large mutations that flip the orientation of sections of DNA, and the presence of inversions has the potential to impact many traits at once. Inversions exist in many organisms, including humans and the fruit fly *Drosophila melanogaster*. We take existing knowledge on *Drosophila* trait variation and identify several inversions which impact many kinds of traits. Approaches such as GWAS can be used to identify the DNA mutations most associated with variation in a trait of interest, but many GWAS methods do not perform well when inversions contribute to variation in phenotype. We show that a common GWAS approach in *Drosophila* is not only unable to find association within inversions, but is overall underpowered. In contrast, we show a different approach is better able to identify mutations within inversions that are potentially associated with fruit fly traits. These findings help scientists studying a wide range of organisms to better understand the phenotypic impact of inversions, and support the broad research of identifying gene associations within these regions of inversion.

## Introduction

Genomic inversions facilitate adaptation by suppressing recombination and generating linkage between many genes and mutations, therefore affecting the genetic basis and evolution of complex traits [1–5]. The adaptive importance of inversions for the evolution of novelty, local adaptation, and speciation is clear from a wide variety of organisms across the tree of life (reviewed in [6–10]). Despite the prevalent role of inversions in evolution, the role of inversions underlying phenotypic variation is often overlooked within association studies [11].

Our understanding of the general importance of inversions in affecting trait variation largely comes from ecological genetics, wherein distinct morphs have been identified in natural populations and subsequently linked to inversions [12]. For instance, conspicuous behavioral, morphological, phenological and life-history variation has been linked to complex inversion polymorphisms in wild populations of birds [13], seaweed flies [14], monkey-flowers [7], and snails [15]. In these cases, and many others [16–18], distinct morphs and their patterns of segregation were first characterized [19–22], prior to identification of inversion genotypes. Therefore, it is less clear if inversions have a major impact on less conspicuous quantitative genetic variation that can be identified through forward mapping approaches, where the goal is to identify the genetic basis of phenotypic variation.

There are two main reasons that forward mapping approaches have potentially missed the impact of inversions on quantitative trait variation. The first reflects the design features of mapping approaches that utilize recombinant populations [23–25]. Because inversions reduce recombination, these mapping panels have intentionally used strains with colinear genomes to facilitate recombination and enable efficient QTL mapping. The second reflects statistical techniques of genome-wide association (GWA) studies of outbred wild or laboratory populations. Modern GWA approaches that factor out population structure may have missed important links between inversions and trait variation because inversions can have a major impact on estimates of population structure and relatedness [26–28]. Thus, the use of population structure or relatedness estimates as a co-factor in GWA analysis may have led to reduced power to detect association with SNPs linked to inversions. Even when inversion-trait associations can be drawn following GWA [15,29–31], it is challenging to identify the specific genetic architecture within inversion driving association due to the high linkage between loci within inversions [32,33]. Therefore, the role of inversions in less conspicuous quantitative genetic variation may have been overlooked in many species.

The fruit fly *Drosophila melanogaster* is an excellent model to assess the importance of inversions on quantitative trait variation. *D. melanogaster* possesses large inversions present at intermediate frequencies worldwide, and flies show evidence of local and rapid adaptation driven by inversions (reviewed in [34]). *D. melanogaster* inversions are known to impact a variety of traits [35–41]. For instance, In(3R)P presence is associated with body size, lifespan, and starvation resistance [37,41], and In(2L)t is associated with behavioral, stress-tolerance, and morphological traits [42–45].

The Drosophila Genetic Reference Panel (DGRP) provides an excellent resource for identifying the effects of cosmopolitan inversions on quantitative variation, and for exploring the role of various GWA methods in discovering associations. The DGRP is a collection of 205 inbred and fully genotyped *D. melanogaster* lines, initially collected from a farmer’s market in North Carolina [46]. The lineages have been inbred, their genomes have been sequenced, and the presence of common inversions has been characterized for each line using a combination of polytene chromosome preparations [26], principal component analysis [47], and PCR [48]. Due to the availability of these resources, DGRP lines have become a common model for phenotyping studies across many traits [49]. To facilitate association studies with the DGRP, several websites have been developed with a standardized mapping approach that factors out inversions and other drivers of relatedness [26,49]. Indeed, GWA approaches that correct for population structure, cryptic relatedness, or inversions account for approximately 60% of DGRP studies from a representative sample of 36 papers (curated dataset;(49)), yet few (35%) report testing for associations with inversions or inversion linked markers (S1 Table).

In our study, we test the ability of different GWA mapping approaches to identify signatures of association with inversions and linked variants. We utilize published studies that measure phenotypic variation in the DGRP. First, we show that several cosmopolitan inversions have large effects on dozens of traits, in that they explain more trait variation than expected by SNPs of comparable frequencies. Next, we explore four genome-wide association strategies that differ in their genetic-relatedness matrices (GRMs) and the treatment of inversions as co-factors, and contrast the real GWA signal for each phenotype to 100 permutations. We generated three types of GRMs (i) using the full genome, (ii) using an LD-thinned genome, (iii) and using a leave-one-chromosome-out (LOCO) approach. In addition, we performed association analysis and permutations using the full-genome based GRM and factored out the effect of inversions following methods outlined in (26,49). We show that the result of the GWA greatly depends on the mapping strategy, and that only the LOCO approach resolves association signals that exceed permutations. We highlight one case study to show that LOCO can identify signals of SNP association with alcohol tolerance within In(2L)t that are missed with the common GWAS method. Finally, we use the output of the LOCO-GWA to test whether SNPs identified as top candidates under the different mapping strategies show different levels of enrichment for signatures of local adaptation, and whether signals of pleiotropy are resolvable at specific loci inside two inversions.

## Materials and methods

### Selection of trait data

We re-analyzed trait data collected on the DGRP [46]. We made use of the DGRPool resource, which has consolidated the phenotypic line averages of inbred DGRP lines from many publications [49]. We used the “curated” data, and removed traits from this dataset that describe genomic features such as genome size or transposon presence, or used less than 75 unique DGRP lines, ending up with 409 unique traits derived from 36 publications (S1 Table). Some traits measure the same or similar phenotype, and thus there is potential for our results to be biased toward more frequently measured phenotypes. Of these 36 studies, 19 of them were also represented in the independent meta-analysis reported in Nunez et al., 2024 [43]. We annotated these traits by classifying each trait into 5 general groups: “Behavior”, “Life-History”, “Morphology”, “Physiology”, and “Stress-resistance” (S1 Table).

### The phenotypic impact of cosmopolitan inversions

We characterized the effect of cosmopolitan inversions In(2L)t, In(2R)NS, In(3L)P, In(3R)K, In(3R)P, and In(3R)Mo on these traits, as these are the inversions considered by the DGRP analysis webtools. For this analysis, we used the inversion classifications provided by (26). The 205 DGRP lines classified here (S2 Table) are the same lineages present in the DGRP genotyping data. For each trait, we used a simple linear model to test the effect of any single inversion using strains that were homozygous for either the inverted or standard allele. We did not include heterozygotes as there is no way to determine the frequency of the inverted allele within DGRP lines marked as heterozygous, nor to know the genotype of any individual that was phenotyped. In addition, we do not consider the impact of multiple inversions simultaneously, nor do we test interactions between inversions because of the relatively low frequency of inversions in the dataset.

For each phenotype, and for each inversion, we compared the results of the linear model with a null model using an ANOVA test, and counted the number of traits with significant association with any of the inversions with p-value < 0.05 (S1 Table). Next, we evaluated whether the extent of association between traits and inversion status is greater than expected relative to other random polymorphisms in the genome. The motivation for this analysis is to test if the inversions have a greater impact than expected by chance. For each inversion, we replicated the above linear modeling using 100 SNPs and small indels randomly selected from those identified at the same frequency as each inversion (±1%), and on the same chromosome arm, but at least 2MB from the inversion breakpoints to avoid areas of highest linkage disequilibrium*.* By comparing the number of traits significantly associated (p < 0.05) with the inversions and random polymorphisms, we can use the 100 random polymorphisms to approximate the effect of a given mutation on trait data expected by chance. Last, we calculated R^2^ (coefficient of determination) for the observed and matched-polymorphism models to ask whether the inversions explain more variation than expected by other comparable SNPs and small indels in the genome. If a trait was significantly associated with an inversion using the linear model results, and if the R^2^ of that model surpassed the 95% quantile of permutations, we assigned that trait to a group of “inversion-associated traits” used in downstream analysis.

One last extension of this analysis was to examine the impact of the ancestry of the DGRP lines on trait line averages. This is important because North American *D. melanogaster* populations, including the DGRP, result from secondary contact and admixture between European and African populations about 150 years ago [50–53]. Using estimates of the proportion of European and African ancestry of each DGRP line from Pool 2015 (53), we created linear models using the same statistical tools as described above. For each phenotype, and each inversion, we evaluated an Ancestry-only model (ancestry as a fixed effect), Inversion-only model (inversion genotype as a fixed effect), and a Full model (both ancestry and inversion genotype as fixed effects) against a null model using ANOVA. Observed models were compared against permutations in which phenotype line averages were shuffled. Last, we repeated this statistical framework, this time comparing the Full model against the Ancestry and Inversion models.

### Principal component analysis of trait data

We used principal component analysis (PCA) to identify broader trends of inversion impact on phenotype. From the original five inversions, we only investigated inversions in which at least 5% of the DGRP samples used were homozygous for the inversion. In this case we focus on two sets of inversion-associated traits: traits associated with In(2L)t or In(3R)Mo (S3 Table). Missing data for any trait were imputed using the *imputePCA* function from missMDA v1.19 [54] using the “regularization” method. This approach uses the average phenotype value for initial imputation, and performs a secondary regularization step. We used the imputed data to conduct PCA using FactoMineR v2.8 [55]. We separated the principal component projections by inversion presence using the inversion genotype of the DGRP lines and then compared the PCs of “inverted” and “standard” groups using Student’s t-test.

### Principal component analysis of genomic data

To understand how inversions impact general patterns of multi-locus genetic variation, we performed a series of PCA on the DGRP SNP and small indel polymorphism data. We used three polymorphism selection strategies for this principal component analysis that mirrors polymorphism selection strategies used for the construction of the GRM (see below). The first version of polymorphism selection (“Full”) used all SNPs and small indels across the autosomes and X chromosome with minor allele frequency (MAF) greater than 5% and sites with missing genotype data in less than 20% of DGRP lines. The second version (“LD”) used SNPs and small indels with MAF > 5% and missing rate < 15%, and a low pairwise linkage disequilibrium (R^2^ < 0.2). To ensure that SNPs were at least 5000 base pairs apart we used the *snpgdsLDpruning* function of the R-package SNPrelate v3.17 [56], with the *slide.max.bp* parameter set to 5000. The third version used a leave-one-chromosome out (“LOCO”) approach [57] that used the same filtering and thinning strategy as the LD-pruning approach but in four parts, each missing one of the main chromosomal arms in order to reduce the effect of an inversion on relatedness within its own chromosomal arm. We used the *snpgdsPCA* function from *SNPrelate* v3.17 [56] for PCA. To quantify the effect of inversions on principal component space, we constructed linear models in the same manner described above, recording the R^2^ of both the linear models and a set of permutations with the lines’ inversion genotype shuffled. We designated a model outcome significant if its R^2^ surpassed 95% of permutations.

### Construction of GRMs

We developed three genomic relatedness matrixes (GRM) to address population structure in different ways. For the “Full” method, we use the GRM matrix that is supplied by the DGRP website and is commonly used in DGRP GWAS studies (http://dgrp2.gnets.ncsu.edu/, last accessed 04/20/2025). This approach uses the VanRaden method [58] to construct a GRM from all SNPs and small indels with a MAF > 0.05 and a missing rate < 20% [26]. For the “LD” method, we used LD pruning using the same parameters that we used for the LD-pruned PCA, described above, and constructed a GRM from the whole genome using the *snpgdsGRM* function in SNPRelate based on the Genome-wide Complex Trait Analysis (GCTA) method [59]. For the “LOCO” method, we generated sub-GRMs, each one drawing from the DGRP genome but ignored one chromosome arm (“2L”, “2R”, “3L”, “3R”, and “X”) and using the same steps as described for the LD-thinned approach.

### GWA analysis

We performed association mapping using mixed-effect models implemented in the R package *GMMAT* v1.3.2 [60]. This approach used either the “Full”, “LD”, and “LOCO” GRMs as a random effect to control for population structure and cryptic relatedness. In addition, we performed a fourth association mapping approach based on the GWA approach developed by Huang *et al.* 2014 [26], which we refer to as the “Factored-out” approach. The Factored-out approach first standardizes each trait by the effects of the inversions by regressing line mean data against inversion status using the model:


trait ~ In(2L)t + In(2R)NS + In(3R)P + In(3R)K + In(3R)Mo

Next, the residuals of this model are used as the trait or association analysis. We used the “Full” GRM with the Factored-out approach to replicate the association model implemented in by Huang et al. 2014 (26) and available on the DGRP online GWA tool (http://dgrp2.gnets.ncsu.edu/).

For each of the four GWA approaches, we compared a “full model” to a “reduced model.” The reduced model is described by the formula:


y ~ Wolbachia + GRM,

where *y* represents the line means for a particular trait (or residuals in the case of the Factored-out model). Wolbachia is an infectious symbiote known to affect aspects of Drosophila fitness [61–63], and is included as a cofactor in standard DGRP GWA approaches. Here we encode Wolbachia infection status as a fixed effect listed as present or absent, based on the tables published in Huang et al., 2014 [26]. GRM is a random effect genetic relatedness matrix. The full model is:


$$\text{y}\;\sim \;{\text{variant}}_{i}\;+\;\text{Wolbachia}\;+\;\text{GRM},$$

where variant_i_ is the fixed effect and an additive representation of the dosage of the *i*^th^ SNP or small-indel reported for the DGRP. We contrasted the full and reduced models using the *glmm.score* function in the GMMAT package (v1.4.2), which filters out all variants with minor allele frequency < 5% and missing data > 15%. In the LOCO approach (57), we split the scoring of the genome into five sections for each of the major chromosomal arms, with each GWA using the sub-GRM constructed without that corresponding region. Our GWA approach scores inverted and non-inverted regions without distinction.

For each trait and GWA method, we conducted 100 permutations by randomly shuffling the trait data prior to fitting the reduced and full models.

### GWA summary statistics

We compared the overall genomic signal from the GWA of each trait using statistics for the observed and permutated GWA models. We partitioned each chromosome into bins based on whether SNPs are inside or outside inversions as defined using coordinates in Corbett-Detig et al. 2012 [48]. For each bin, we calculated the proportion of SNPs with a p-value less than 10^-5^ as “hits”, a common p-value threshold in DGRP studies [64–70]. We also calculated the genome inflation factor (GIF) as the ratio of the median observed variant-wise Χ^2^ values within the bin divided by the expected median Χ^2^ with 1 degree of freedom. We compared the summary statistics for the observed data to the statistics from the permutations for each trait and GRM method, reporting the proportion of traits where the statistic exceeds the 95th percentile of the trait’s permutation-based distribution.

### Enrichment tests

We tested if GWA hits identified via different GRM approaches prioritize SNPs that are potentially subject to temporally or spatially variable selection as identified by BayPass v2.1 [71]. We used data from the DEST dataset to obtain allele frequencies from *D. melanogaster* population*s* sampled across the North American East Coast [72], and Charlottesville, Virginia across multiple years [43]. We identified polymorphisms that are more differentiated than expected given population structure (XtX* outliers) and tested association between variants and environmental variables after correcting for population structure. Using this framework, we identified differences in allele frequencies between populations (XtX*) and Bayes Factor (BF) of association with environmental variables. We used latitude for the association of East Coast variants and maximum temperature two weeks prior to collection for Charlottesville variants [43]. We ran the software five times and report the mean statistic per SNP [73]. We generated a null distribution of XtX* and BF using the POD (Pseudo-observed Data) framework for 10-times the number of SNPs as the observed data, ran BayPass with five replicate iterations, and calculated empirical p-values for XtX* and BF using these POD simulations.

We identified the level of enrichment between top GWA variants and top BayPass variants. We identified the top hits within each GWA study by identifying the 500 hits with the lowest P-value, and top XtX* and BF variants as those that surpass 95% of the corresponding distribution from the simulated POD data. We computed Fisher’s Exact test by contrasting the odds that top association hits for any trait are enriched for top XtX* and BF hits. We compared these Fisher’s Exact test odds-ratios to odds-ratios constructed in the same way using the permuted GWA.

### COLOC analysis

We tested if inverted regions of the genome are likely to have pleiotropic effects on phenotypic variation using the *coloc.abf* function from coloc v5.2.3 [74]. By treating the top principal component projections as dimensionality-reduced traits, we sought to identify regions in the genome with a shared association with multiple inversion-linked traits. We used the Factored-out and LOCO GWA frameworks to score the impact of SNPs genome-wide on the PC1 and PC2 loadings for the In(2L)t and In(3R)Mo associated traits. We identified areas of colocalized signal on PC1 and PC2 using a sliding window analysis across the genome with window size 10Kb and step size 5Kb, and compared the SNP GWA data using *coloc.abf*(). This analysis identified regions of SNPs likely associated with the traits differentiated along only PC1, only PC2, or regions of SNPs with a colocalized association across PC1 and PC2.

## Results

### Cosmopolitan inversions impact phenotypic variation

**Fig 1:**
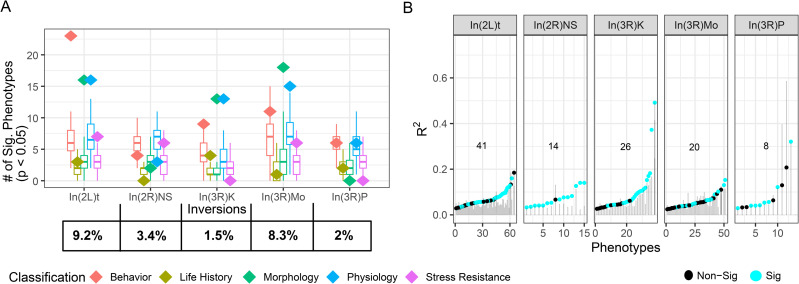
Cosmopolitan inversions have a major impact on trait variation. **A)** Diamonds, colored by trait category, indicate the number of traits significantly affected by inversion presence at p < 0.05, overlaying the same-frequency models shown with box plots. The percentage of lines homozygous for each inversion within the lineages tested is given at the bottom. **B)** The proportion of variation explained by each inversion (R^2^) for traits significantly associated at p < 0.05 with each inversion, compared against the distribution of corresponding same-frequency models in grey. Statistically significant inversion model values that surpass the null distribution are colored cyan. The number in each panel of B is the count of phenotypes where R^2^ exceeds permutations and traits that are significantly associated with the inversion with p < .05.

To study the role of inversions on genetically based trait variation in *D. melanogaster*, we re-analyzed data from publications that measured trait variation in the DGRP and were curated in the DGRPool database [49]. We analyzed 409 traits, categorizing them into five groups: morphological, life-history, stress resistance, physiological, and behavioral (S1 Table). We found that In(2L)t, In(3R)Mo, and In(3R)K are associated with more traits than expected given SNPs of the same frequency (Fig 1A). In(2L)t is especially associated with many behavioral traits including startle response, sleep, and movement, while In(3R)K and In(3R)Mo are associated with morphological traits such as femur and abdomen size (S1 Table). We found that the inversions explain ~10% of the variation in these traits, and that dozens of traits are explained better by inversion status than expected from random polymorphisms in the genome (Fig 1B). Signals of association with the inversions are not explainable by variation in African ancestry among DGRP lines (S1 Fig).

### Principal component analysis of traits associated with In[2L]t and In[3R]Mo

**Fig 2:**
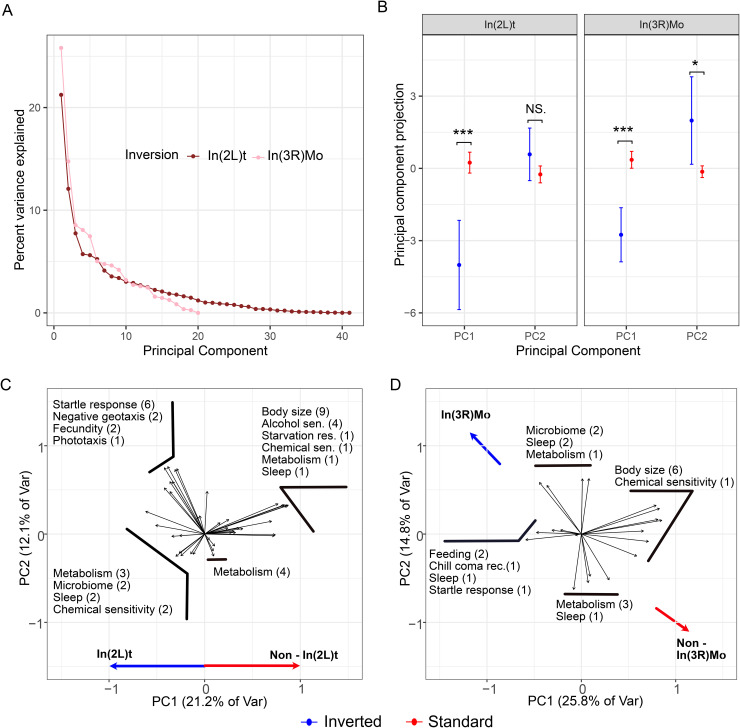
Principal component analysis of In(2L)t and In(3R)Mo. **A)** A scree plot showing the variance explained by principal components, colored by their associated inversion. **B)** The effect of In(2L)t and In(3R)Mo genotype on PC1 and PC2 projection values. Points represent the mean and confidence intervals represent two standard errors. **C)** PC loading values for the traits significantly impacted by In(2L)t. Labels are aggregated to show similar traits together, e.g., “Sleep (2)” corresponds to two sleep traits. Variance explained by each PC is given on the axis title. **D)** PC loading values for the traits significantly impacted by In(3R)Mo. In B, C, and D colors represent the homozygous genotype.

To better understand the impact of inversions on phenotypic variation, we performed PCA on the traits that are associated with In(2L)t and In(3R)Mo and also explain more variation than expected by chance (In(2L)t: n = 41, In(3R)Mo: n = 20; Fig 1). The top two principal components (PC1 and PC2) explain over one third of the trait variation for both In(2L)t and In(3R)Mo (Fig 2A). Therefore, we restrict further analysis to these two principal components. In(2L)t significantly loads onto PC1 (t-test, t = -4.38, df = 16.68, p = 4.29e-4) of its associated trait set, while In(3R)Mo significantly loads onto both PC1 (t-test, t = -5.18, df = 18.03, p = 6.21e-5) and PC2 (t-test, t = 2.27, df = 15.54, p = 0.038; Fig 2B) of its associated trait set. For the In(2L)t PCA, traits like body size and ethanol sensitivity have positive loadings on PC1 and traits like startle response and negative geotaxis have negative loading. Lines homozygous for In(2L)t have lower values of PC1, thus have higher startle response and higher activity levels (Fig 2C), among other differences (S3 Table). For the In(3R)Mo PCA, traits like body size have positive loadings on PC1 and traits like feeding and chill coma recovery have negative loading. Lines homozygous for In(3R)Mo have lower values of PC1, thus have lower body size (Fig 2D), among other differences (S3 Table).

### Controlling the effect of inversions on PC and GRM space

**Fig 3:**
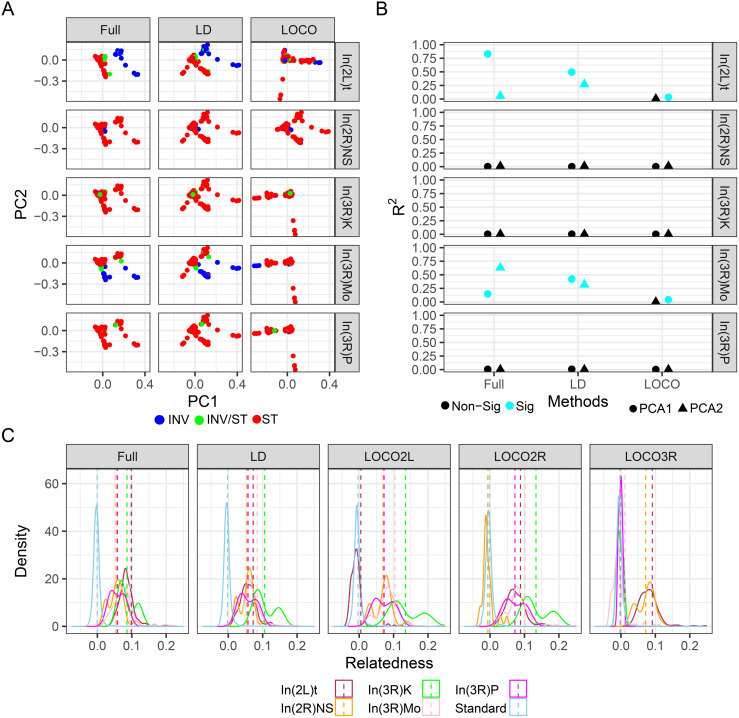
Inversions influence population genetic structure and relatedness. **A)** The first and second genomic PCs for each sample colored by the genotype of that sample. **B)** The R^2^ values for models comparing PC1 and PC2 to inversion, colored by which values exceed a distribution of permutations. **C)** The distribution of pairwise relatedness values between each of the samples, indicated by a solid line colored by genotype of the samples and split across different methods, with a dotted line indicating means.

The cosmopolitan inversions of *D. melanogaster* have been previously shown to have an impact on genome-wide patterns of genetic variation as summarized by principal components and genetic relatedness matrices [27,28,75]. Therefore, we tested if different polymorphism selection strategies can mitigate this impact. As previously reported [26], PCA of the “Full” genome shows that inversions strongly impact PC space. In(2L)t primarily impacts PC1_Full_ (F _1,179_ = 870, p = 2.89e-70) and In(3R)Mo primarily impacts PC2_Full_ (F _1,195_ = 331, p = 5.54e-44, Fig 3A). The “LD” polymorphism-set slightly reduces the impact on PC1_LD_ for In(2L)t (F_1,179_ = 175, p = 2.89e-28) and the impact on PC2_LD_ for In(3R)Mo (F_1,195_ = 90.71, p = 6.93e-18, Fig 3A). In contrast, PCA of the LOCO genome shows a sharply reduced impact of In(2L)t on PC1_LOCO_ (F _1, 179_ = 5.84, p = 0.017) and In(3R)Mo on PC2_LOCO_ (F _1,195_ = 0.02, p = 0.88, Fig 3A).

We calculated the proportion of variation in the genetic PC1 and PC2 that is explained by inversion status, and contrasted that to a null distribution made via 100 permutations. We found for the Full and LD methods, In(2L)t and In(3R)Mo explained more variation for PC1 and PC2 than expected by chance (Fig 3B), with In(2L)t explaining the most variance within PC1 using the Full method and less using the LD method, while In(3R)Mo explained the most variance for PC2 within the Full method and less within the LD method. Meanwhile, within the LOCO method each inversion explains a near zero amount of variance for PC1 or PC2, and PC2 is no longer significantly impacted by either inversion (Fig 3B). For PC3 and PC4, the R^2^ of each of the principal component ~ inversion genotype models were near zero, indicating there is little correlation between inversion genotype and these principal components (S2 Fig).

To identify how the presence of inversions impacts patterns of relatedness, we compared the relatedness of the DGRP lines using different polymorphism selection strategies. Across all approaches, relatedness is low within standard genotype lines with no cosmopolitan inversions (Fig 3C). This replicates observations in Huang et al. 2014 (26). However, when using the Full and LD-thinned approaches, relatedness is noticeable between lines that are both homozygous for any given inversion. In contrast, the LOCO approach on a given chromosome can drive relatedness for homozygous inverted lines near zero while still accounting for inversions on the other chromosomal arms (Fig 3C).

### The LOCO approach can better capture signals for inversion-associated traits

**Fig 4:**
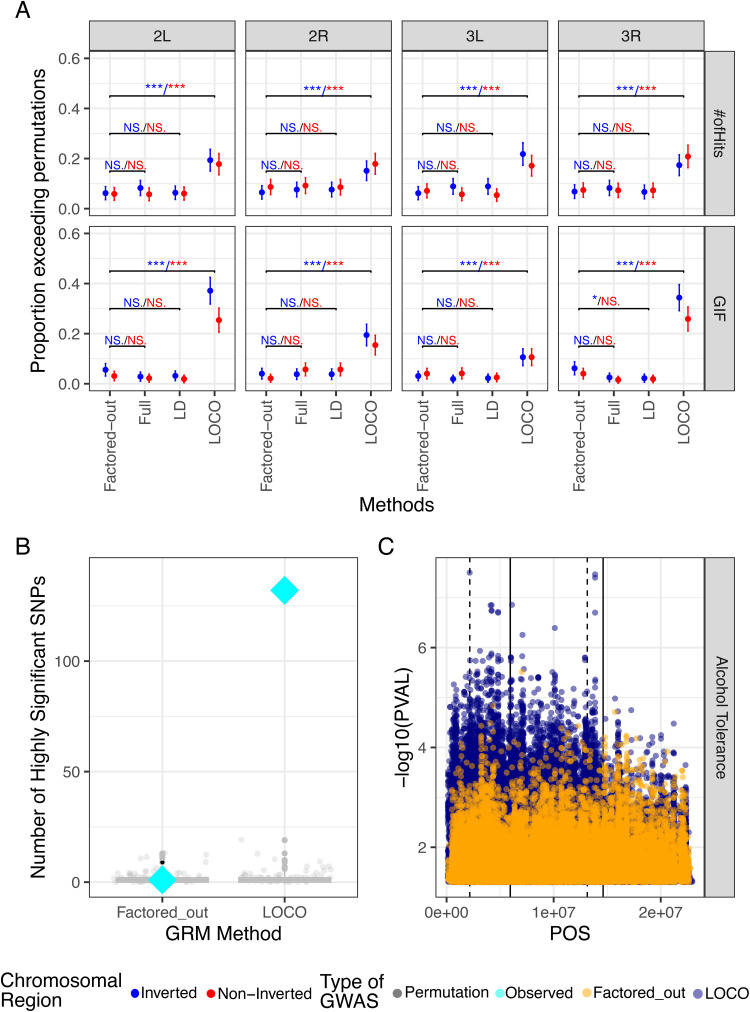
The LOCO approach can better capture signal genome-wide. **A)** The proportion of significant hits and GIF for each GWAS output are compared across each method and colored by location relative to inversions. The proportion of traits that exceed their corresponding permutations is given on the y axis, along with binomial confidence intervals. The color of the significance annotation refers to the chromosomal region under comparison. (* = p < 0.05, ** = p < 0.01, *** = p < 0.001, NS. = p >= 0.05) **B)** The number of significant hits (p < 1e-5) on 2L for male ethanol tolerance for LOCO and Factored-out GWA is compared against permutations. Color refers to observed or permuted GWAS results. **C)** The strength of association of variants with male ethanol tolerance is shown across 2L. The left-most solid line indicated the *Gdph* gene, and the right-most indicates the *Adh* gene. The dotted lines indicated the breakpoints of In(2L)t. Color refers to the GWA approach used.

After characterizing the impact of different GRM methods and presence of inversion in the DGRP data, we tested the strengths and weaknesses of the four GWA strategies (Factored-out, Full, LD, LOCO) on the magnitude of association signal. We compared the summary statistics from the observed trait GWA against permutations to see how many traits identified more signal than expected by chance. Using the Factored-out approach, we found that about 6% of traits have “hit-counts” that exceeds the largest 95% of the null distribution generated by permutation (Fig 4). In other words, the Factored-out approach largely fails to identify more associations than would be expected by random chance, as 6% is about the number of traits that would surpass permutations as false positives. We found that the Full and LD-thinned approaches also fail to identify many more significant associations than expected given the permutations. The LOCO method surpasses permutation significantly more often than the Factored-out method within inverted regions (Fisher’s Exact Test - FET, 2L: p = 9.47e-9, 2R: p = 5.17e-4, 3L: p = 6.66e-10, 3R: p = 1.61e-7), as well as outside the inverted region (FET, 2L: p = 1.2e-9, 2R: p = 7.71e-4, 3L: p = 1.61e-5, 3R: p = 9.19e-13) (Fig 4A). Similarly, the GIF of LOCO surpasses permutation significantly more often than the Factored-out method within inverted regions (Fisher’s Exact Test - FET, 2L: p = 7.05e-45, 2R: p = 3.14e-12, 3L: p = 7.42e-6, 3R: p = 2.21e-29), as well as outside the inverted region (FET, 2L: p = 1.59e-19, 2R: p = 1.38e-13, 3L: p = 2.35e-5, 3R: p = 1.15e-21: (Fig 4A). However, this increase in GIF via LOCO is not uniform across the chromosome. The GIF of traits scored with LOCO is significantly higher when scored in inverted regions than non-inverted regions on both 2L (FET, p = 4.34e-8) and on 3R (FET, p = 0.014).

As a case study to highlight how statistical methods impact signals of association in GWA studies, we focused on male alcohol tolerance as measured by Morozova et al., 2015 [76]. This phenotype is correlated with In(2L)t (Fig 4B and S1 Table) and is among the phenotypes that our analyses focused on. Performing GWA on male ethanol tolerance using the factored-out and LOCO approaches yields starkly different results. The Manhattan plot of the GWA results for the LOCO method shows a strong signal of association with SNPs near In(2L)t, a signal that is absent using the Factored-out approach. Indeed, when comparing the GWA signal from the real data to permutations, we observe that the LOCO method yields far stronger signals of association than permutations, while the factored-out method does not exceed permutations. Intriguingly, the strongest signal of association for male ethanol tolerance is found at a SNP near the distal breakpoint of In(2L)t, whereas signals of association at the closely linked alcohol dehydrogenase (*Adh*) gene is not apparent (Fig 4C).

### Enrichment tests

**Fig 5:**
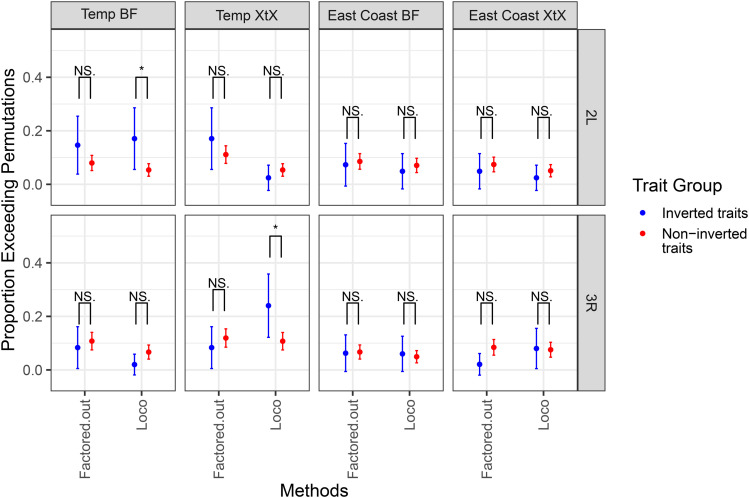
Enrichment of GWAS hits with environmentally associated SNPs. We used two population sets: a temporal set collected in Charlottesville, and a latitudinal set collected across the North American east coast. BF (Bayes Factor) outliers are top loci associated with temperature (Charlottesville temporal population set) and latitude (East Coast population set). XtX outliers are the most strongly differentiated SNPs through time (Charlottesville) or space (East Coast). We report the proportion of traits for whom the enrichment between BayPass SNPs and real GWA SNPs exceeds the 95% largest enrichment with the GWA permutations. Error bars represent 95% binomial confidence intervals. Color indicates whether the traits are associated with inversion. (* = p < 0.05, ** = p < 0.01, *** = p < 0.001, NS. = p >= 0.05).

To compare the utility of the four approaches to identify biologically meaningful loci, we characterized the ability of LOCO and Factored-out methods to identify loci thought to be important for local adaptation. Using estimates of allele frequencies of *D. melanogaster* collected across seasons and across latitudes [72, 77], we used the Baypass [71] software to identified SNPs that are more strongly differentiated across the North American east coast, or within Charlottesville, VA through time (XtX* outliers). In addition, we identified the strength of association between SNPs and latitude for the East Coast samples and between SNPs and temperature in the two weeks prior to sampling for the Charlottesville samples. To understand which methods could successfully identify enrichment within inverted regions, we compared enrichment signals between inversion associated and not-associated traits. There was a significant jump in enrichment between GWA hits and candidate SNPs that have large BF association with maximum temperature for the LOCO method on 2L (FET, p = 0.019), but not for hits derived from the Factored-out method (Fig 5). Correspondingly, there was an increase in enrichment between GWAS hits and differentiation across max temperatures for the LOCO methods on 3R (FET, p = 0.011) but not for the Factored-out method (Fig 5). There was no difference reported within the East Coast enrichments with GWA hits between inverted and non-inverted associated traits.

### COLOC enrichment within the genome

**Fig 6:**
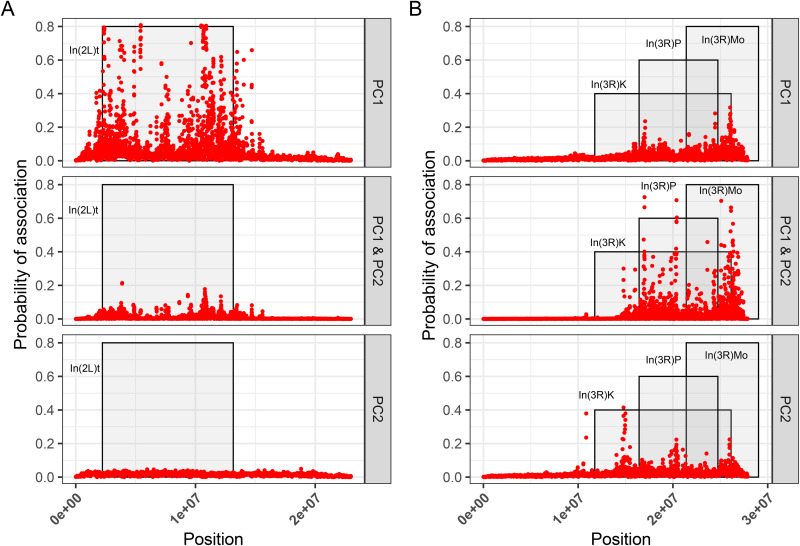
The LOCO method reveals areas of likely colocalized association for inversion-related traits. **A)** Results of a sliding window analysis examining enrichment between SNPs scored using LOCO for PC1 and PC2 of In(2L)t. The y-axis shows the likelihood of association, and the x-axis shows position on the genome. The grey shaded regions show the zone of cosmopolitan inversions on the chromosome arm. **B)** Same analysis as in A, but for traits associated with In(3R)Mo. D likelihood.

To compare the ability of GWA approaches to identify potentially pleiotropic associations of inversions with orthogonal multivariate-traits, we calculated the probability that regions of the genome share polymorphisms that affect multi-dimensional traits (co-localization). Using the top principal component projections from Fig 2 as dimensionality reduced traits, we scored the effect of SNPs and small indels genome-wide on PC1 and PC2 using the LOCO and Factored-out GWA approaches. We followed with a sliding window analysis to identify loci within the genome are that likely associated with traits differentiated along only PC1, the traits differentiated along only PC2, or for both PC1 and PC2. With the LOCO method, we identified variants within the In(2L)t inverted regions and near the breakpoints that have high association likelihood with PC1 of the In(2L)t-linked traits (Fig 6A) while there was little association on other chromosome arms (S3 Fig). In contrast, for In(3R)Mo areas of likely association were identified across 3R for both PC1 and PC2, with the peaks aligning with other inversion breakpoints on 3R (Fig 6B) and several peaks observed on other chromosomes (S4 Fig). Notably, the peaks of the highest likelihood of association differed between PC1 and PC2, suggesting that distinct loci within the inverted regions influenced different sets of traits. In contrast, SNPs scored using the Factored-out method failed to capture any signal of likely association with either PC1, PC2, or both (S5 Fig).

## Discussion

Genomic inversions can simultaneously influence multiple traits and provide a mechanism for adaptation. Associations between inversions and phenotypic variation have been identified across the tree of life, along with evidence that natural selection acts upon these genomic features [6,7,8]. Here, we find that inversions within *D. melanogaster* impact a suite of diverse traits (Figs 1A, 2C, and 2D), and specific statistical methods are better equipped to map associations between inversion-linked loci and these traits. Inversions should be considered areas of interest, rather than areas to be skipped over within association studies, as their presence here is shown to explain large parts phenotypic variation (Figs 1B and 2B), and strongly impacts estimates of relatedness derived from polymorphism data (Fig 3). We illustrate that different GWA approaches have different power in the number and strength of associations they can identify (Fig 4). Compared to several commonly used methods, the LOCO approach is better able to identify variants linked to inversions that are associated with key traits. We show that these variants are enriched for loci that could underlie local adaptation and that these variants are likely pleiotropic (Figs 5, 6A, and 6B).

Previous work has linked *D. melanogaster* inversions to phenotypic variation, implicating these mutations in changes to body size, wing size, longevity, and more [35–41]. Despite these known impacts, only 13 out of the 36 publications aggregated here report any test for association between inversions and their trait(s) of study (S1 Table). Here we reexamined the impact of inversions on a large body of diverse trait data, and show that inversions like In(3R)Mo and In(2L)t significantly affect more traits than would be expected by chance, including inversion-trait associations not previously identified (S1 Table and Fig 1). In(3R)Mo varies across latitudinal clines in multiple continents [3], and the overlapping inversion In(3R)P is thought to facilitate local adaptation [34,36,78,79]. In our analysis, In(3R)Mo is likely more enriched than In(3R)P due to its higher frequency. Here we confirm that inversions on chromosome 3R impact body size (Fig 2D). Similarly, highlighting the association between In(2L)t and activity (Figs 1A and 2C) provides new avenues for investigation for the ongoing link to this inversion and seasonal adaptation [43,80]. Taken together, we show that inversion presence explains considerable trait variance for specific traits (Figs 1B and 2B), indicating these inversions should be a major factor in consideration for association studies.

Inversions provide a challenge for association studies, as the increased linkage disequilibrium and relatedness within inverted samples can elevate the false discovery rate [28,75]. Many modern GWA techniques thus seek to mitigate the impact of relatedness by using top principal components as cofactors [81] or factoring our relatedness using GRMs as a random-effect [82]. The Factored-out approach described here employs such methods, using genome-wide GRMs and additionally factors out the effect of inversions prior to genome-wide association mapping. Of the studies we analyzed, 21/36 used this method or an equivalent for GWA with the DGRP (S1 Table). However, we report that only about 6% of GWA using this method find more hits than from random permutations (Fig 4), indicating a lack of power and a potentially high false-positive rate amongst many published DGRP studies. Other GWA methods, such as thinning the relatedness matrix for linkage disequilibrium, fare little better (Fig 4). In contrast, LOCO is designed to identify association when there is high LD within the genome, by avoiding proximal contamination between highly linked SNPs while still partially accounting for population structure [57]. Recent association studies have used LOCO methods of establishing relatedness while investigating association studies within inversions and other areas of high LD [15,83]. For example, Calboli *et al.,* 2022 established an association between an agriculturally relevant trout disease and an inversion used a LOCO method, but not with their accompanying “Full” genome method [84]. Here we provide new evidence that LOCO has the potential to outperform other methods at identifying association signal, especially when inversions are present in mapping populations (Fig 4).

To highlight the differences in GWA signal between the Factored-out and LOCO approach we focused on alcohol tolerance. Alcohol tolerance is an ecologically relevant trait for *D. melanogaster*, given their preferred habitat of rotting and fermenting fruit [85, 86]. While the yeast that colonizes rotting fruit provides an important food source for larval [87, 88] and adult flies [89], the ethanol produced as a byproduct of fermentation can act as a strong selective pressure [90, 91]. A principal *Drosophila* enzyme involved in metabolizing ethanol is *Adh*, and genetic variation in the *Adh* gene and protein has been a focus of empirical population genetics since the inception of the field [92–94]. Notably, the *Adh* gene harbors two allozyme variants in *D. melanogaster* (Fast and Slow; [95]), generated by a single non-synonymous polymorphism (K192T; [92]), and these variants display clinal patterns of allele frequency change consistent with the action of spatially [96, 97] and temporally varying selection [98]. As a consequence, the role of the *Adh* polymorphism in explaining heritable genetic variation in ethanol tolerance has been a long-standing area of research [99]. Recent work using genetically engineered variants clearly shows that enzymatic activity of these alleles contributes to genetic differences in adult ethanol tolerance [100]. However, a DGRP GWA study of adult ethanol tolerance using the factored-out approach [76] did not recover any signal of association at *Adh,* or other genes with natural enzyme polymorphism linked to ethanol tolerance such as *Gdph* and *Mdh1* [101–103]. Morozova *et al.* attribute the lack of signal at these and other classical ethanol tolerance genes to limited statistical power or physiological buffering.

The *Adh* gene resides on chromosome 2L and is several megabases outside of the boundaries of In(2L)t. Yet, polymorphism at *Adh* is tightly linked to In(2L)t [104, 105]. This linkage could result from recombination interference that is generated by inversion heterozygotes [106]. In addition, it is possible that epistatic selection is maintaining linkage disequilibrium between *Adh* and In(2L)t [107–109]. Epistasis is plausible given that *Gdph* resides within the boundary of In(2L)t and is tightly linked to the inversion and *Adh* [44, 110]. There is some empirical support for epistasis from selection experiments [101] and wild population surveys [110], but this hypothesis lacks support from transgenic approaches [103]. Nonetheless, linkage disequilibrium between *Adh* and In(2L)t, coupled with possible epistatic interactions between these loci, could impact signals of association for ethanol tolerance.

In our analysis, analysis choices for GWA has a strong impact on signals of association with male ethanol tolerance **(Fig 4B and 4C)**. Using the factored-out approach, we do not recover any signal of association at *Adh* **(Fig 4C)** similar to the original study [76]. In addition, using the factored-out approach, there is no signal of association greater than we expect by chance (Fig 4B). However, the LOCO approach yields much more interesting signals of association. Notably, SNPs tightly linked to In(2L)t are strongly associated with male ethanol tolerance, consistent with our ANOVA results **(Fig 1)**. The strongest signal of association in the genome is a SNP that resides immediately adjacent to the inversion breakpoint, and another noticeable peak of association is immediately adjacent to the *Gdph* locus (Fig 4C). In the Factored-out approach, there is not a strong signal generated by *Adh*, *Gdph*, or In(2L)t. While the causal roles of In(2L)t, *Gdph*, and *Adh* on male ethanol tolerance in the DGRP remain unclear, a major candidate locus is missed when performing GWA using the Factored-out approach. GWA approaches that avoid proximal contamination may therefore be important in GWA, especially when inversions or other large structural variants are present.

Incorporating data on clinal allele variation indicates that the LOCO method may provide advantages in identifying loci involved in inversion-mediated adaptation. Several studies have indicated that In(2L)t could mediate seasonal adaption [80,111], and one study suggested that behavioral traits could contribute to rapid seasonal adaptation [79]. We investigated the enrichment between the top GWA hits and an independent set of environmentally relevant alleles. We discovered that top GWA hits within In(2L)t are enriched for seasonally associated loci, and that GWA hits within In(3R)Mo are enriched with loci that differentiate over latitudinal clines (Fig 5). The overlap of environmentally varying alleles and top GWA loci can be identified from LOCO-based GWA, but not from the Factored-out approach (Fig 5).

We identify differences in the ability of GWAS approaches to identify regions within the genome that are associated with multiple traits (pleiotropy). The ability of inversions to pleiotropically effect multiple traits has already been noted in salmonids [84, 112], and mice [6,113–115]. In principal component analysis, different aspects of body size can load onto the top principal component, reflecting some unifying aspect of body size development [116]. In contrast, phenotypic variation across principal components can reflect a different degree of pleiotropy across orthogonal traits. Thus, we characterized areas of high association between PC1 and PC2 of the inversion linked trait sets to illustrate such pleiotropy using a colocalization test. Only the LOCO approach identifies that the areas of highest association with multiple inversion-linked traits are near their corresponding inversions, as one might expect (Fig 6A and 6B). However, within the inverted regions there are peaks of higher likelihood of association, similar to the finding in Nunez et al., 2024 of peaks of SNP-phenotype enrichment within In(2L)t [43]. Peaks of association with PC1 of In(3R)Mo may indicate loci relevant to traits such as body size, while a peak for PC2 may indicate different loci relative to traits such as metabolic storage and sleep, and the peaks for both PCs indicate areas of likely pleiotropic effect (Fig 2D). While LOCO can aid in identifying these areas of likely association, this signal cannot be recapitulated using the Factored-out approach (S5 Fig).

Inversions have the potential to be a fruitful area of investigation within association studies. Despite the evidence across taxa that inversions can influence many classes of traits (6,7,78), inversions are sometimes presented as a statistical hindrance [28,75]. Efforts such as the creation of popular mapping populations from largely co-linear genotypes [23–25], and the use of multiple methods to factor out inversion presence within the DGRP [26] represent steps to account for these mutations. To be clear, the phenotyping and association studies from the DGRP and other mapping populations have produced many important and foundational insights. However, models that remove or ignore inversions miss a valuable opportunity. Methods like LOCO offer tools toward building association studies to identify relevant loci linked to inverted regions (84,85). Inversions can play a significant role in the traits of humans and across many forms of life [117–120]. Improving our ability to connect inversion to traits will motivate future work to better understand how these complex mutations contribute to trait regulation and formation.

## Supporting information

S1 Fig The addition of ancestry proportion does not remove the broad impact of inversion genotype on phenotype. **A)** The number of phenotypes with significant associations is shown as diamonds for the Ancestry and Inversion model, as well as for a Full model that uses both ancestry and inversion genotype as fixed effect. A set of paired 100 permutations of each model is shown as a box and whisker plot. Results are split across five cosmopolitan inversions, and colored by trait classification. **B)** The same plot as in (A), now showing a comparison between the Full and Ancestry models, as well as the Full and Inversion models.
(DOCX)

S2 Fig Genomic principal components PC3 and PC4 have little correlation with inversion genotype. **A)** The third and fourth genomic PCs for each sample colored by the genotype of that sample. **B)** The R^2^ values for models comparing PC3 and PC4 to inversion, colored by which values exceed a distribution of permutations.
(DOCX)

S3 Fig Signal of loci association with In(2L)t is mostly adjacent to the inversion. The same results of the association study using the LOCO method from Fig 6 are shown across the genome, showing the likelihood of a SNP’s association with PC1, PC2, or both from the In(2L)t PCA analysis.
(DOCX)

S4 Fig Signals of loci association with In(3R)Mo is elevated on 3R. The same results of the association study using the LOCO method from Fig 6 are shown across the genome, showing the likelihood of a SNP’s association with PC1, PC2, or both from the In(3R)Mo PCA analysis.
(DOCX)

S5 Fig The Factored-out method fails to identify areas of likely association. **A)** Results of a sliding window analysis examining enrichment between SNPs on 2L scored using Factored-out for PC1 and PC2 of In(2L)t, the y- axis shows the strength of enrichment and the x-axis shows position on the genome. Grey shaded region show the zone of cosmopolitan inversions on the chromosome arm. **B)** Same analysis as in A, but considering chromosome arm 3R and inversion In(3R)Mo.
(DOCX)

S1 Table Phenotype metadata. This table includes the background information for the DGRP studies and individual phenotypes that passed quality control and are used throughout this publication. For each phenotype the name and doi of the originating study is listed. Additionally, the table includes metadata relating to the number of DGRP lines with data for each trait, and the relationship of each trait to cosmopolitan inversions.
(XLSX)

S2 Table The inversion genotype of DGRP lines. This table indicates the genotype for the five cosmopolitan inversions examined in this study, for each of the 205 lineages genotyped within the DGRP. This data is directly taken from the DGRP website (http://dgrp2.gnets.ncsu.edu/, last accessed 04/20/2025).
(XLSX)

S3 Table The principal components of inversion-related traits. This table further describes the sets of traits found to be significantly associated with In(2L)t or In(3R)Mo, showing the top 5 principal components for the PCA run on each of the two sets of traits.
(XLSX)
