# Supplementary material for: Cosmopolitan inversions have a major impact on trait variation and the power of different GWAS approaches to identify associations
Source: PLoS Genet. 2026 Jan 5;22(1):e1012012. doi: 10.1371/journal.pgen.1012012 (PMC12818957; doi:10.1371/journal.pgen.1012012)
Supplement: S1 Fig — A) The number of phenotypes with significant associations is shown as diamonds for the Ancestry and Inversion model, as well as for a Full model that uses both ancestry and inversion genotype as fixed effect. A set of paired 100 permutations of each model is shown as a box and whisker plot. Results are split across five cosmopolitan inversions, and colored by trait classification. B) The same plot as in (A), now showing a comparison between the Full and Ancestry models, as well as the Full and Inversion models. (DOCX) [file pgen.1012012.s001.docx]

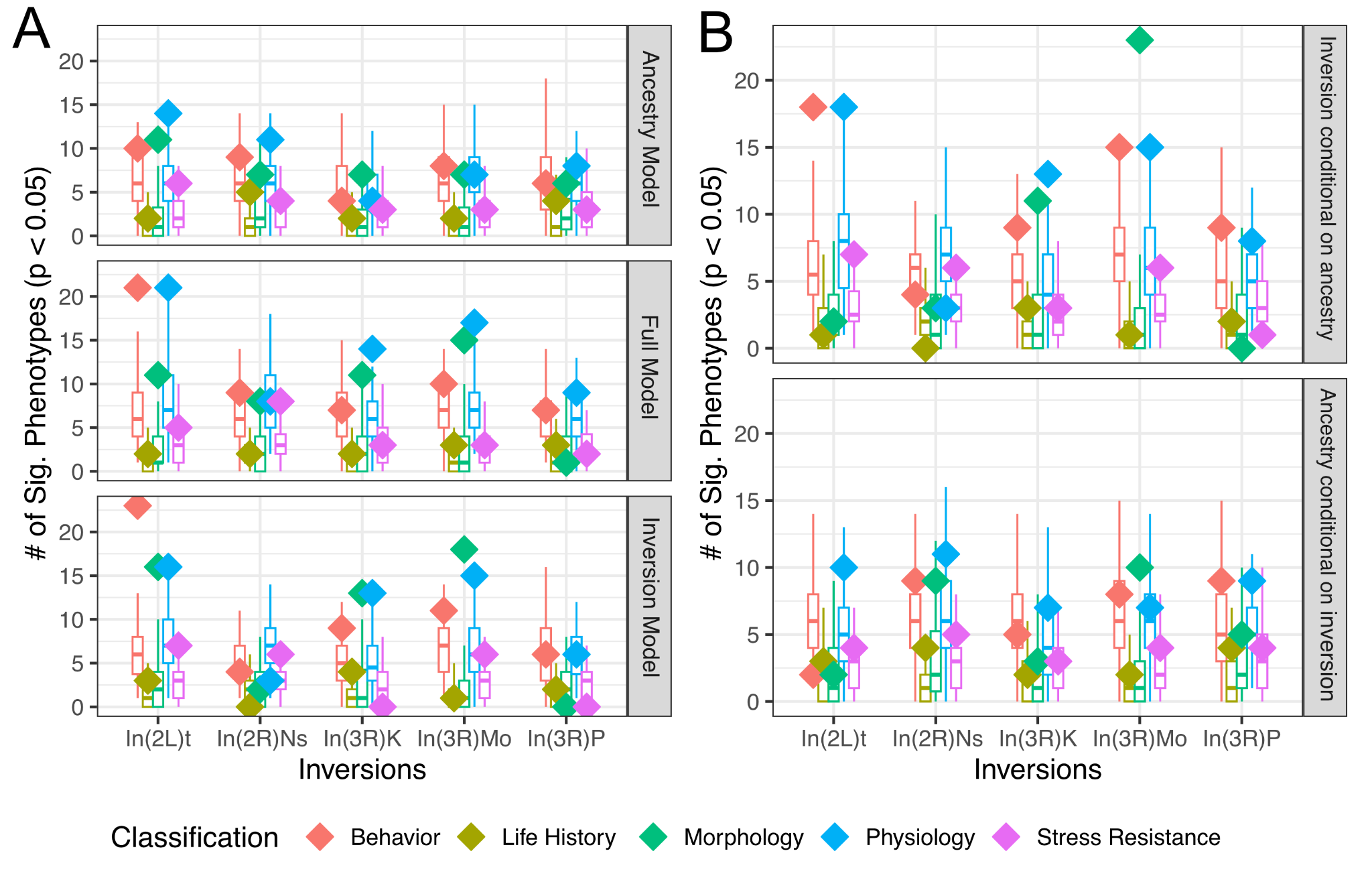


**S1 Fig: The addition of ancestry does not remove the broad impact of inversion genotype on phenotype. A)** The number of phenotypes with significant associations is shown as diamonds for the Ancestry and Inversion model, as well as for a Full model that uses both ancestry and inversion genotype as fixed effect. A set of paired 100 permutations of each model is shown as a box and whisker plot. Results are split across five cosmopolitan inversions, and colored by trait classification. **B)** The same plot as in A, now showing a comparison between the Full and Ancestry models, as well as the Full and Inversion models.
