# Supplementary material for: Cosmopolitan inversions have a major impact on trait variation and the power of different GWAS approaches to identify associations
Source: PLoS Genet. 2026 Jan 5;22(1):e1012012. doi: 10.1371/journal.pgen.1012012 (PMC12818957; doi:10.1371/journal.pgen.1012012)
Supplement: S2 Fig — A) The third and fourth genomic PCs for each sample colored by the genotype of that sample. B) The R2 values for models comparing PC3 and PC4 to inversion, colored by which values exceed a distribution of permutations. (DOCX) [file pgen.1012012.s002.docx]

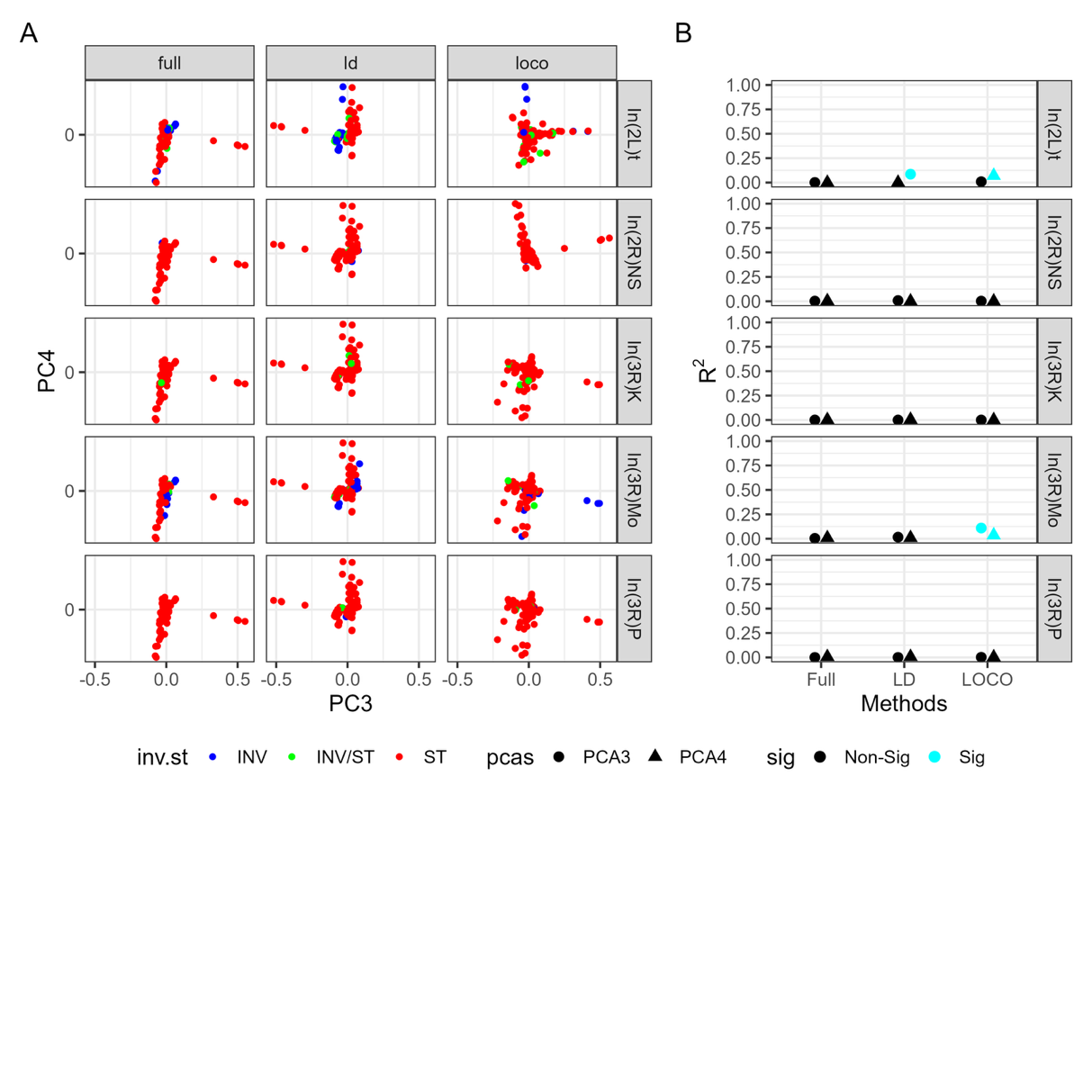


**S2 Fig.** Genomic principal components PC3 and PC4 have little correlation with inversion genotype. **A)** The third and fourth genomic PCs for each sample colored by the genotype of that sample. **B)** The R^2^ values for models comparing PC3 and PC4 to inversion, colored by which values exceed a distribution of permutations.
