# Supplementary material for: Cosmopolitan inversions have a major impact on trait variation and the power of different GWAS approaches to identify associations
Source: PLoS Genet. 2026 Jan 5;22(1):e1012012. doi: 10.1371/journal.pgen.1012012 (PMC12818957; doi:10.1371/journal.pgen.1012012)
Supplement: S4 Fig — The same results of the association study using the LOCO method from Fig 6 are shown across the genome, showing the likelihood of a SNP’s association with PC1, PC2, or both from the In(3R)Mo PCA analysis. (DOCX) [file pgen.1012012.s004.docx]

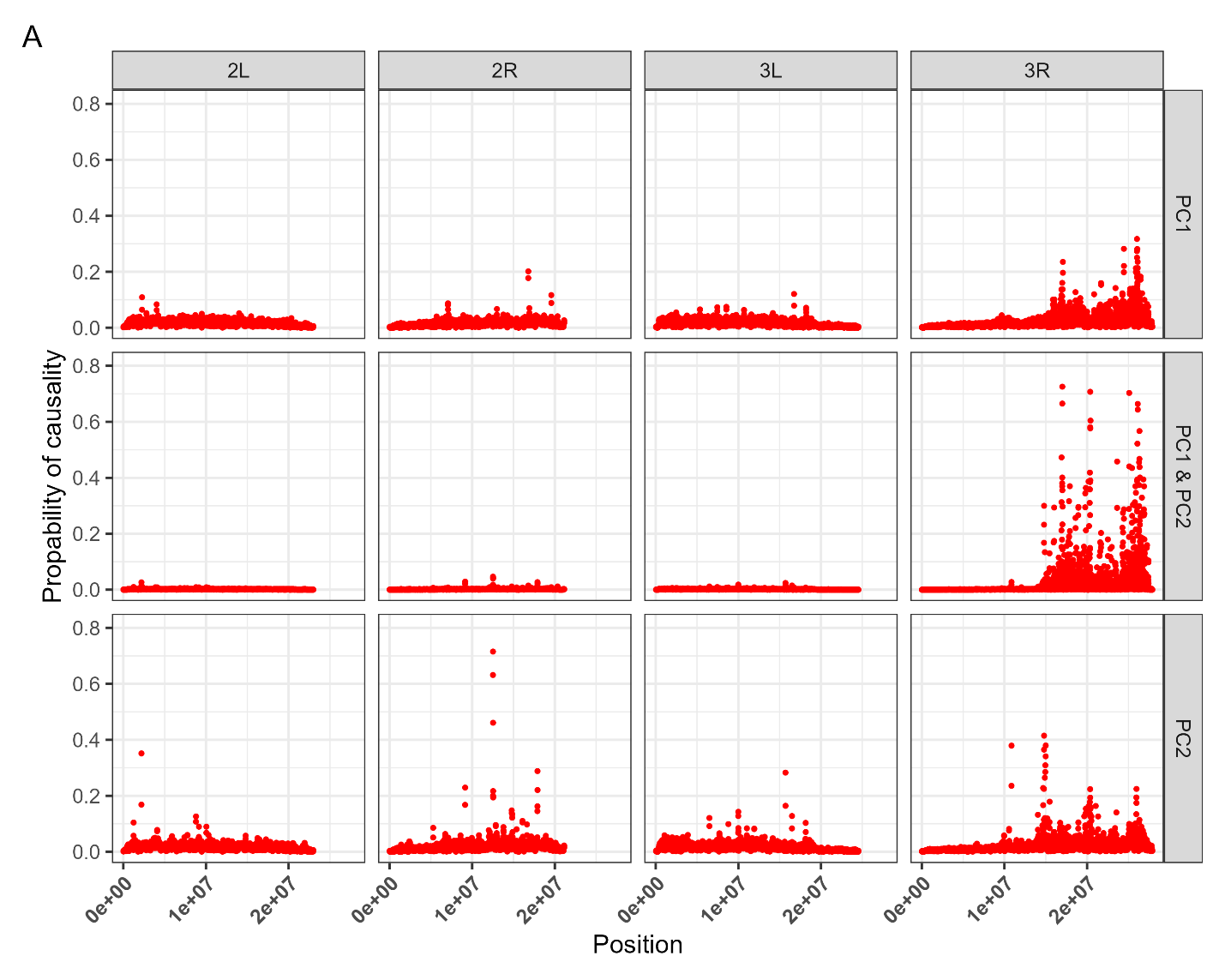


**S4 Fig.** Signal of loci association with In(3R)Mo is elevated on 3R. The same results of the association study using the LOCO method from Figure 6 are shown across the genome, showing the likelihood of a SNP’s association with PC1, PC2, or both from the In(3R)Mo PCA analysis
