# Supplementary material for: Cosmopolitan inversions have a major impact on trait variation and the power of different GWAS approaches to identify associations
Source: PLoS Genet. 2026 Jan 5;22(1):e1012012. doi: 10.1371/journal.pgen.1012012 (PMC12818957; doi:10.1371/journal.pgen.1012012)
Supplement: S5 Fig — A) Results of a sliding window analysis examining enrichment between SNPs on 2L scored using Factored-out for PC1 and PC2 of In(2L)t, the y- axis shows the strength of enrichment and the x-axis shows position on the genome. Grey shaded region show the zone of cosmopolitan inversions on the chromosome arm. B) Same analysis as in A, but considering chromosome arm 3R and inversion In(3R)Mo. (DOCX) [file pgen.1012012.s005.docx]

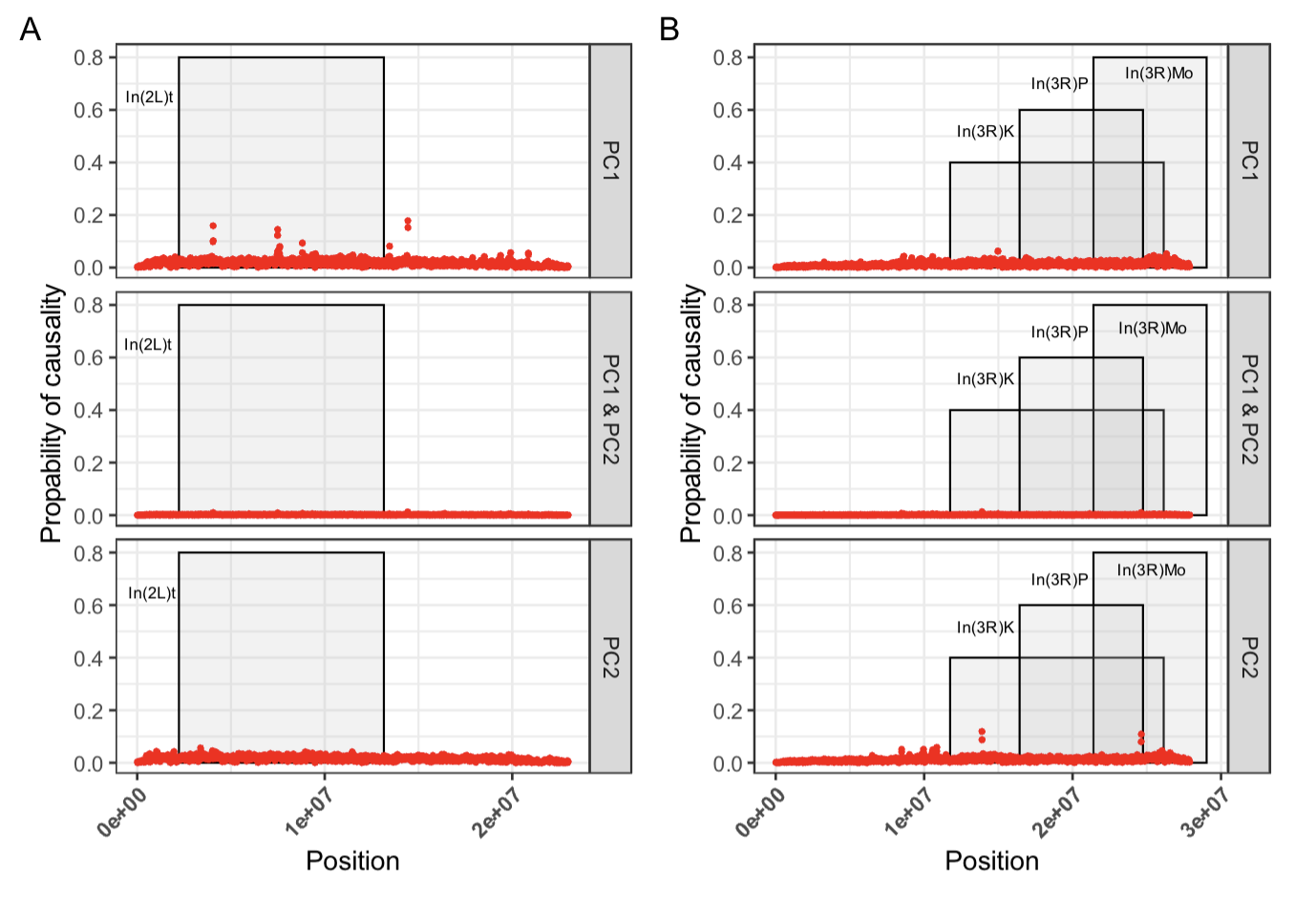


**S5_Fig.** The Factored-out method fails to identify areas of likely association. **A)** Results of a sliding window analysis examining enrichment between SNPs on 2L scored using Factored-out for PC1 and PC2 of In(2L)t, the y- axis shows the strength of enrichment and the x-axis shows position on the genome. Grey shaded region show the zone of cosmopolitan inversions on the chromosome arm. **B)** Same analysis as in A, but considering chromosome arm 3R and inversion In(3R)Mo.
